# Current Evidence and Future Perspective of Accuracy of Artificial Intelligence Application for Early Gastric Cancer Diagnosis With Endoscopy: A Systematic and Meta-Analysis

**DOI:** 10.3389/fmed.2021.629080

**Published:** 2021-03-15

**Authors:** Kailin Jiang, Xiaotao Jiang, Jinglin Pan, Yi Wen, Yuanchen Huang, Senhui Weng, Shaoyang Lan, Kechao Nie, Zhihua Zheng, Shuling Ji, Peng Liu, Peiwu Li, Fengbin Liu

**Affiliations:** ^1^First College of Clinic Medicine, Guangzhou University of Chinese Medicine, Guangzhou, China; ^2^Department of Spleen-Stomach and Liver Diseases, Traditional Chinese Medicine Hospital of Hainan Province Affiliated to Guangzhou University of Chinese Medicine, Haikou, China; ^3^Department of Gastroenterology, First Affiliation Hospital, Guangzhou University of Chinese Medicine, Guangzhou, China

**Keywords:** artificial intelligence, machine learning, deep learning, early gastric cancer, endoscopy

## Abstract

**Background & Aims:** Gastric cancer is the common malignancies from cancer worldwide. Endoscopy is currently the most effective method to detect early gastric cancer (EGC). However, endoscopy is not infallible and EGC can be missed during endoscopy. Artificial intelligence (AI)-assisted endoscopic diagnosis is a recent hot spot of research. We aimed to quantify the diagnostic value of AI-assisted endoscopy in diagnosing EGC.

**Method:** The PubMed, MEDLINE, Embase and the Cochrane Library Databases were searched for articles on AI-assisted endoscopy application in EGC diagnosis. The pooled sensitivity, specificity, and area under the curve (AUC) were calculated, and the endoscopists' diagnostic value was evaluated for comparison. The subgroup was set according to endoscopy modality, and number of training images. A funnel plot was delineated to estimate the publication bias.

**Result:** 16 studies were included in this study. We indicated that the application of AI in endoscopic detection of EGC achieved an AUC of 0.96 (95% CI, 0.94–0.97), a sensitivity of 86% (95% CI, 77–92%), and a specificity of 93% (95% CI, 89–96%). In AI-assisted EGC depth diagnosis, the AUC was 0.82(95% CI, 0.78–0.85), and the pooled sensitivity and specificity was 0.72(95% CI, 0.58–0.82) and 0.79(95% CI, 0.56–0.92). The funnel plot showed no publication bias.

**Conclusion:** The AI applications for EGC diagnosis seemed to be more accurate than the endoscopists. AI assisted EGC diagnosis was more accurate than experts. More prospective studies are needed to make AI-aided EGC diagnosis universal in clinical practice.

## Introduction

Gastric cancer is ranked as the third leading cause of death from cancer worldwide (1). Most gastric cancers are diagnosed at advanced stages because their symptoms and signs tend to be inconspicuous and non-specific, leading to an overall poor prognosis, whereas in the case of early detection, the 5–years survival rate can exceed 90% (2–4). Endoscopic examination is still considered the most effective method for EGC detection (5). However, early gastric cancer (EGC) is particularly difficult to identify since it usually exhibits a subtle elevation or depression with faint redness, which is likely recognized as normal mucosa or gastritis. In addition, the invasion depth within the gastric wall is also hard to predict. Ten studies involving 3,787 patients who received an upper gastrointestinal endoscopy examination revealed an 11.3% miss rate of upper gastrointestinal cancers up to 3 years before diagnosis (6). A meta-analysis involving 2,153 lesion images showed that the area under the receiver operating characteristic curve (AUC) for the diagnosis of EGC using white light imaging (WLI) endoscopy was only 0.48 (7).

**Figure 1:**
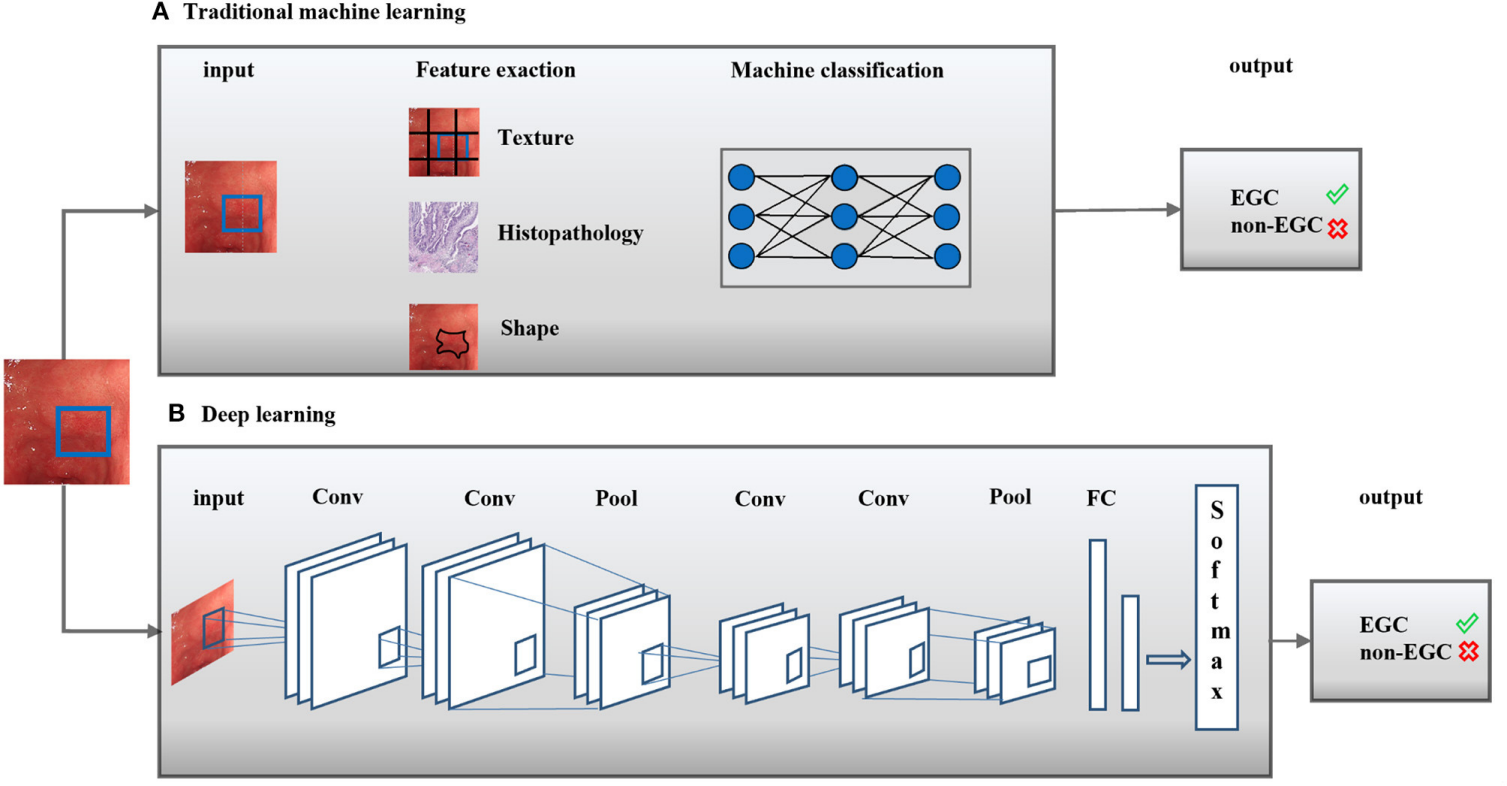
Artificial intelligence methods in medical imaging. Artificial intelligence (AI) methods for a typical classification task were shown. Two classical methods comprise traditional machine learning **(A)** and deep learning **(B)**. Conv, Convolutional layer; Pool, Pooling layer; FC, receiver operating characteristic curve; EGC,: Early gastric cancer.

In the past decade, the application of artificial intelligence (AI) in medicine has attracted extensive attention. AI-assisted endoscopic diagnosis is a hot spot of research. AI refers to the capacity of a computer to execute a task associated with intelligent beings, such as the “learn” function that mimics the cognitive ability of human beings (8). AI subfields contain machine learning and deep learning (Figure 1). Machine learning, a term originally created by Arthur Samuel in 1959, is a field of computer science, whereby a system is able to develop the ability to “learn” from the input data without a certain program (9). Common machine-learning methods in classification model training comprise ensemble trees, decision trees, support vector machines, k-nearest neighbors, etc. (10).

Deep learning, which was initially applied in the image processing field in 1998, refers to the application of layers in non-linear processing based on machine learning algorithms used for feature extraction and transformation (11). Neural networks, similar to the human brain, particularly mimic closely interconnected neurons to recognize patterns, extract features or “learn” things about the input data to predict a result (12). Different model training paradigms, such as scaled-conjugate gradient, Levenberg-Marquardt and Bayesian regularization, have been termed “neural networks” (13). Several computer aided detection (CAD) algorithms for automatic early gastric cancer detection have been recommended for images from standard endoscopes. The performance improvements of original image classification models mainly depend on visual features and large-scale datasets, which are difficult to implement in EGC detection models. Although the invasion depth in EGC is defined differently, visual characteristics such as textures, colors, shapes, and regions are similar.

To date, the existing data on the diagnostic value of AI for EGC diagnosis are scattered. Jin et al. (14) reviewed the current studies on AI application for gastric cancer, while the definite diagnostic ability of AI application for EGC was still unclear. The aim of this study was to systematically summarize the recent available studies on the diagnostic accuracy of AI on EGC diagnosis to address the current status of this area and discuss future perspectives.

## Methods

### Search Strategy and Study Selection

Electronic databases (PubMed, Web of Science, EMBASE, and the Cochrane Library) were searched from initiation to November 2020 using presupposed search terms. The following medical subject terms and keywords were used: “endoscopy,” “Endoscopic Diagnosis,” “early gastric cancer,” “artificial intelligence,” “computer-assisted diagnosis,” “Deep learning,” and “Convolutional neural network.” The full texts of potentially appropriate studies were then reviewed after the screenings of citations and abstracts exported from the electronic databases. The search strategy was shown as follows: (1) (artificial intelligence [Title/Abstract]) OR (computer-assisted diagnosis [Title/Abstract]) OR (Deep learning [Title/Abstract]) OR (Convolutional neural network [Title/Abstract]) (2) (endoscopy [Title/Abstract]) OR (Endoscopic Diagnosis [Title/Abstract]) OR (early gastric ancer [Title/Abstract]) (3) (1) AND (2).

### Study Eligibility Criteria

The eligible studies fulfilled the following criteria: (1) the study was a diagnosis test about AI application in endoscopy for EGC diagnosis. Diagnosis test included AI detection of EGC from other gastric disease or distinguishment of invasion depth; (2) the absolute numbers of true-positive, false-negative, true-negative, and false-positive observations for EGC diagnosis were reported directly or were able to be calculated; (3) the study provided clear information about the database and number of images; (4) the study clearly described the CAD or CNN algorithms and the process applied in the EGC diagnosis.

### Data Extraction

Two reviewers (Jiang X. T., Wen Y.) independently extracted information, including the author, publication year, region, study type, endoscopy modality, algorithm gold standard and dataset, and used the quality assessment of diagnostic accuracy studies-2 instrument to assess the quality of the study (15). Divergence was resolved through discussion and the involvement of the third reviewer (Li P. W.).

### Statistical Analysis

Stata, version 14.2 (StataCorp, College Station, TX) was used for all statistical analyses. Graphpad Prism 8.2.1 was used to delineate the histogram. The TP, FP, FN, and TN observations of each study were input, and the pooled sensitivity and specificity with the 95% confidence intervals (CIs) for EGC diagnosis with AI were thus calculated. The forest plot was delineated. The inconsistency index (I2) test was used to evaluate the heterogeneity between studies using sensitivity (16). A fixed-effects model would be used with a I2 value <50%. More than 50% of the *I*^2^ values indicated significant heterogeneity. Under this situation, a random-effects model would be applied, and subgroup analysis and influence analysis were performed. A summary receiver operating characteristic (ROC) curve was plotted (17). The area under the curve (AUC) was calculated to estimate the diagnostic accuracy. When the AUC reaches 1.0, it suggests an excellent performance diagnostic test, while if the AUC approaches 0.5, it suggests a poor performance test. Publication bias was evaluated by the Deeks test.

## Result

### Literature Search and Characteristic of Studies

A total of 3,714 studies were retrieved after the search. After removing duplicated studies and excluding improper studies, 17 studies were reserved in this systematic analysis. While Ling et al. (18) distinguished differentiated and undifferentiated type EGC with a sensitivity and specificity of 88.6 and 78.6%, thus was finally excluded in our meta-analysis. A total of 16 studies were finally included in the meta-analysis according to the PRISMA flowchart (Supplementary Figure 1). Three studies were from Korea, eight studies were from Japan, four studies were from China, and one was from Pakistan. Nine studies used white light endoscopy (WLE) images to establish a training dataset, five studies used narrow band imaging (NBI) images, and two used both WLE and NBI images. Four studies distinguished the invasion depth of EGC. Seven studies compared the diagnostic ability of AI with endoscopists. Two studies applied video to train the dataset. No prospective studies were carried out currently. The general algorithm methods were Visual Geometry Group-16 (VGG-16), ResNet-50, GoogLeNet, Single Shot MultiBox Detector (SSD), Inception neural network and Support vector machines (SVM) classifier. Yoon et al. applied two kinds of algorithm models in his study. The basic characteristics of the included studies and the risk of bias using the Quality Assessment of Diagnostic Accuracy Studies (QUADAS-2) tool are presented in Table 1 and Supplementary Figure 2.

**Table 1:** Basic characteristic of the included studies.

	**Year**	**Nation**	**Study type**	**Endoscopy for training**	**Image type**	**Format**	**Processing image size**	**DL**	**Algorithm**	**Affiliated tools**	**Gold standard**	**Training database**	**Endoscopist involvement**	**Real-time**
Yoon et al. (19)	2019	Korea	Retrospective	WLE	Image	Not mentioned	Not mentioned	CNN	VGG-16(20)	Grad-CAM	WHO classification of Tumors (21), Japanese classification (22)	Gangnam Severance Hospital, Yonsei University College of Medicine, Korea	No	No
Cho et al. (23)	2019	Korea	Retrospective	WLE	Image	JPEG	1,280 × 640 pixels	CNN	Inception-Resnet-v2	SGD	Histopathology	Endoscopically biopsied or EMR/ESD lesions from Chuncheon and Dongtan Sacred Heart Hospitals,Korea	Yes	No
Sakai et al. (24)	2018	Japan	Retrospective	WLE	image	Not mentioned	224 × 224 pixels	CNN	GoogLeNet(25)	No	Histopathology	Not mentioned	No	No
Horiuchi et al. (26)	2019	Japan	Retrospective	ME-NBI	Image	Not mentioned	224 × 224 pixels	CNN	GoogLeNet	No	Histopathology	Cancer Institute Hospital, Ariake, Koto-ku, Japan	No	No
Lan et al. (27)	2019	China	Retrospective	ME-NBI	Image	Not mentioned	299 × 299 pixels to 512 × 512 pixels	CNN	Inception-v3	Keras deep learning framework	Revisited Vienna classification of gastrointestinal epithelial neoplasia(28)	Four hospitals in four areas of Zhejiang province	Yes	No
Toshiaki et al. (29)	2018	Japan	Retrospective	WLE, Chromoendoscopy and NBI	image	Not mentioned	300 × 300 pixels	CNN	SSD(30)	No	Japanese classification	Cancer Institute Hospital Ariake, Japan, Tokatsu Tsujinaka Hospital, Japan and Tomohiro Institute of Gastroenterology and Proctology, Japan, Lalaport Yokohama Clinic, Japan	No	No
Yan et al. (31)	2019	China	Retrospective	WLE	image	Not mentioned	299 × 299 pixels	CNN	ResNet50(32)	No	Japanese classification	Endoscopy Center of Zhongshan Hospital, China	Yes	No
Kanesaka et al. (33)	2017	Japan	Retrospective	ME-NBI	image	Not mentioned	40 × 40 pixels	CAD	SVM classifier	No	pathology-proven EGCs resected by ESD	Ethics Committee of the Osaka International Cancer Institute	No	No
Wu et al. (34)	2018	China	Retrospective	WLE, NBI, BLE	video	Not mentioned	224 × 224 pixels	CNN	VGG-16, ResNet-50	No	Histopathology	Renmin Hospital of Wuhan University, China	Yes	Yes
Miyaki et al. (35)	2013	Japan	Retrospective	magnifying endoscope	image	Not mentioned	1,280 × 1024 pixels	CAD	SVM classifier	No	Histopathology	Hiroshima University Hospital	No	No
Ikenoyama et al. (36)	2020	Japan	Retrospective	WLE	image	Not mentioned	300 × 300 pixels	CNN	SSD	SGD	Histopathology	Cancer Institute Hospital Ariake, Tokatsu-Tsujinaka Hospital, Tada Tomohiro Institute of Gastroenterology and Proctology, Lalaport Yokohama Clinic, Japan	Yes	No
Ali et al. (37)	2018	Pakistan	Retrospective	Chromoendoscopy	Image	Not mentioned	Not mentioned	CAD	SVM classifier	G2LCM descriptors	Not mentioned	Public data-set at the Portuguese Institute of Oncology	No	No
Bun-Joo et al. (38)	2020	Korea	Retrospective	WLE	Image	JPEG	480 × 480 pixels	CNN	Inception-ResNet-v2 and DenseNet- 161	Class activation map (CAM)	Histopathology	Chuncheon Sacred Heart Hospital	No	No
Horiuchi et al. (39)	2020	Japan	Retrospective	ME-NBI	Video	Not mentioned	224 × 224 pixels	CNN	GoogLeNet	SGD	Histopathology	Lesions initially treated with ESD at the CancerInstitute Hospital	Yes	No
Ueyama et al. (40)	2020	Japan	Retrospective	ME-NBI	Image	Not mentioned	224 x 224 pixels	CNN	ResNet50	SGD	Japanese Classification	Department of Gastroenterology, Juntendo University School of Medicine	No	No
Zhang et al. (41)	2020	China	Retrospective	WLE	Image	Not mentioned	Not mentioned	CNN	ResNet34	DeepLabv3 structure	Histopathology	Gastric cases admitted to Peking University People's Hospital	Yes	Yes

*WLE, White Light Endoscopy; NBI, Narrow Band Imaging; BLI, blue-laser imaging; WHO, World Health Organization; SVM, support vector machine; SSD, Single Shot MultiBox Detector; CNN, Convolutional Neural Network, CAD, Computer-aided diagnosis; Grad-CAM, gradient-weighted class activation mapping; VGG-16, Visual Geometry Group-16, SVM, Support vector machines, SGD, Stochastic gradient descent*.

### Diagnostic Performance of AI on EGC Diagnosis

**Figure 2:**
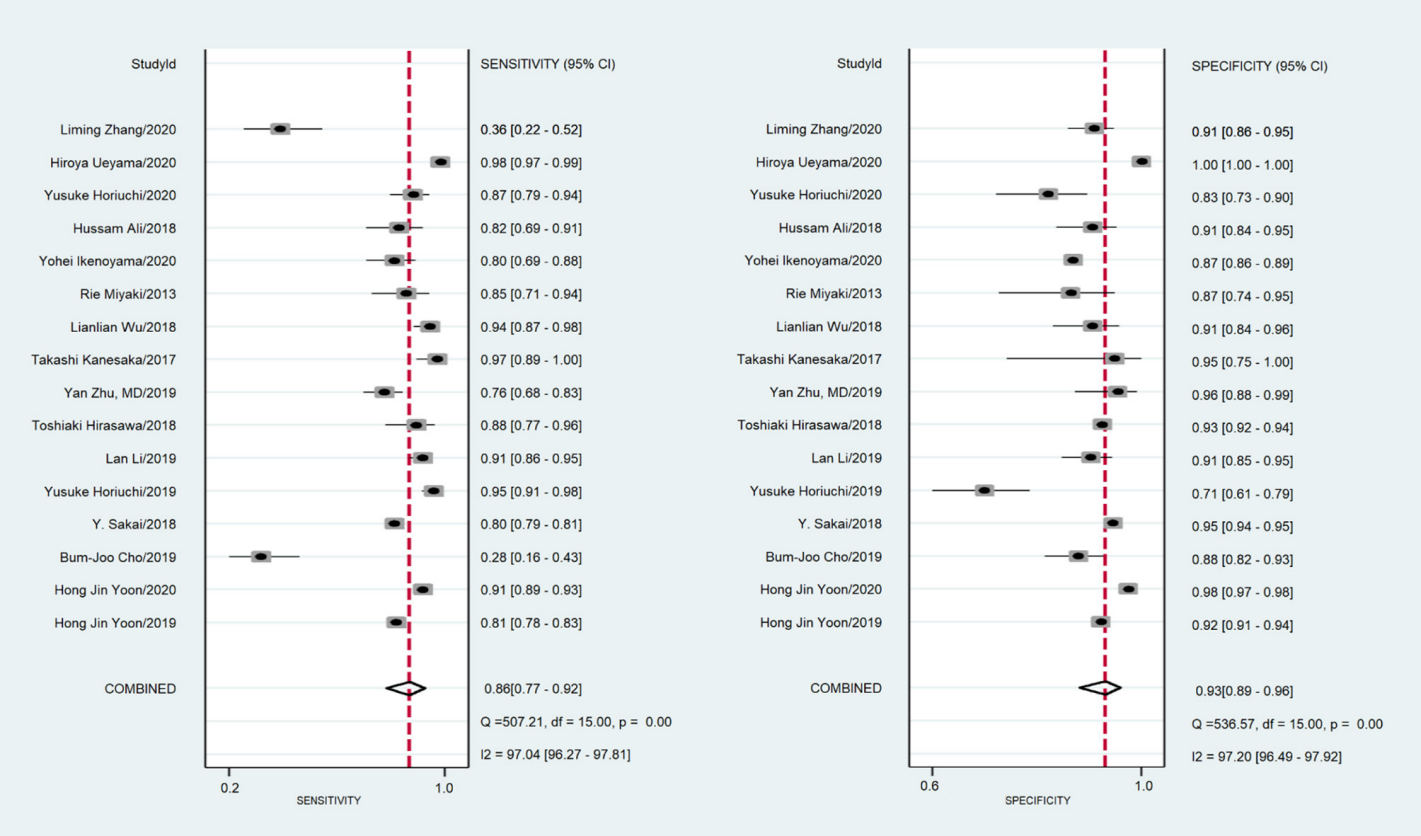
The forest plot of pooled sensitivity and specificity of AI detection on EGC. The pooled sensitivity was 86% (95% CI, 77–92%) and specificity was 93% (95% CI, 89–96%).

**Figure 3:**
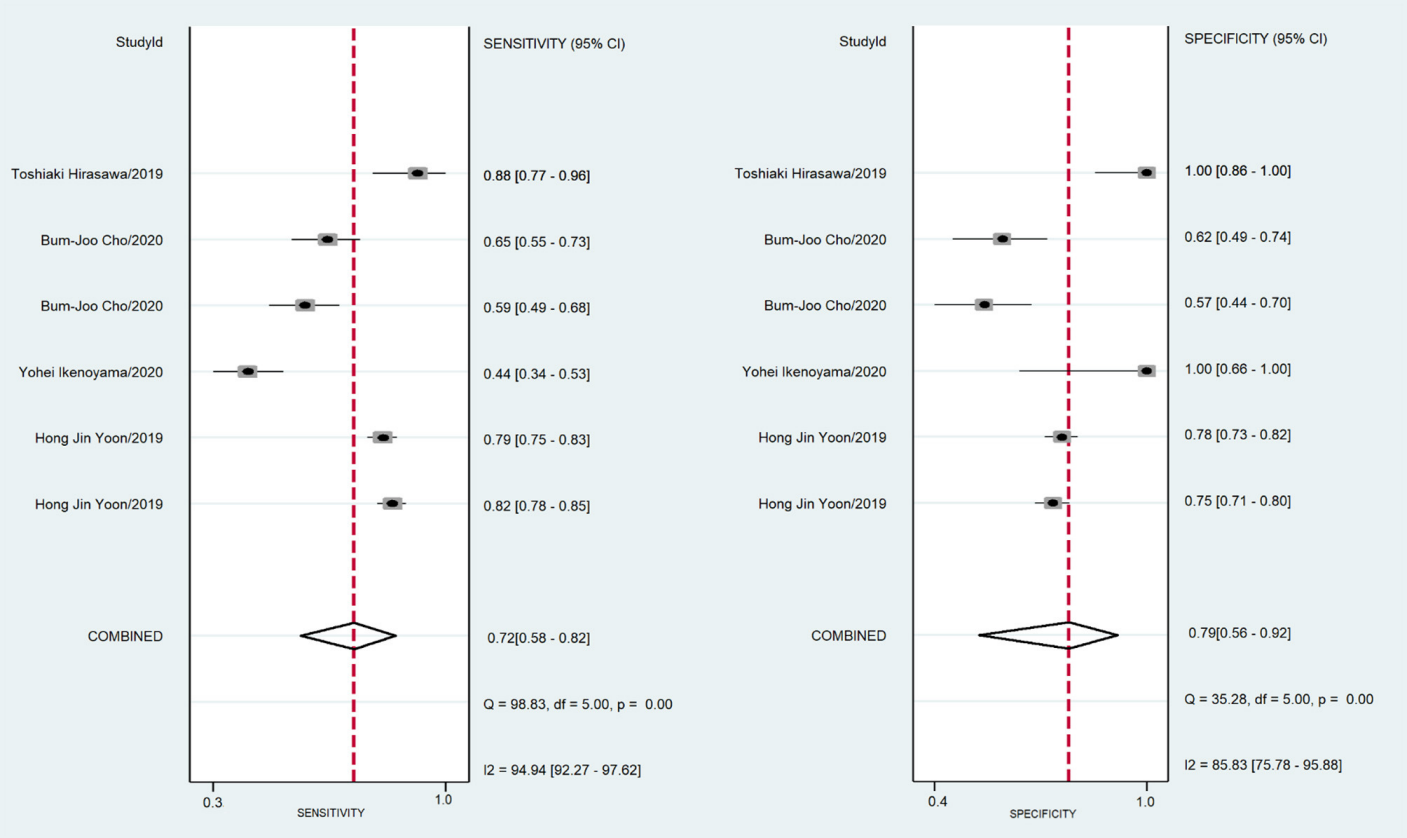
The forest plot of pooled sensitivity and specificity of AI distinction depth on EGC. The pooled sensitivity was 72% (95% CI, 58–82%) and specificity was 79% (95% CI, 56–92%).

**Figure 4:**
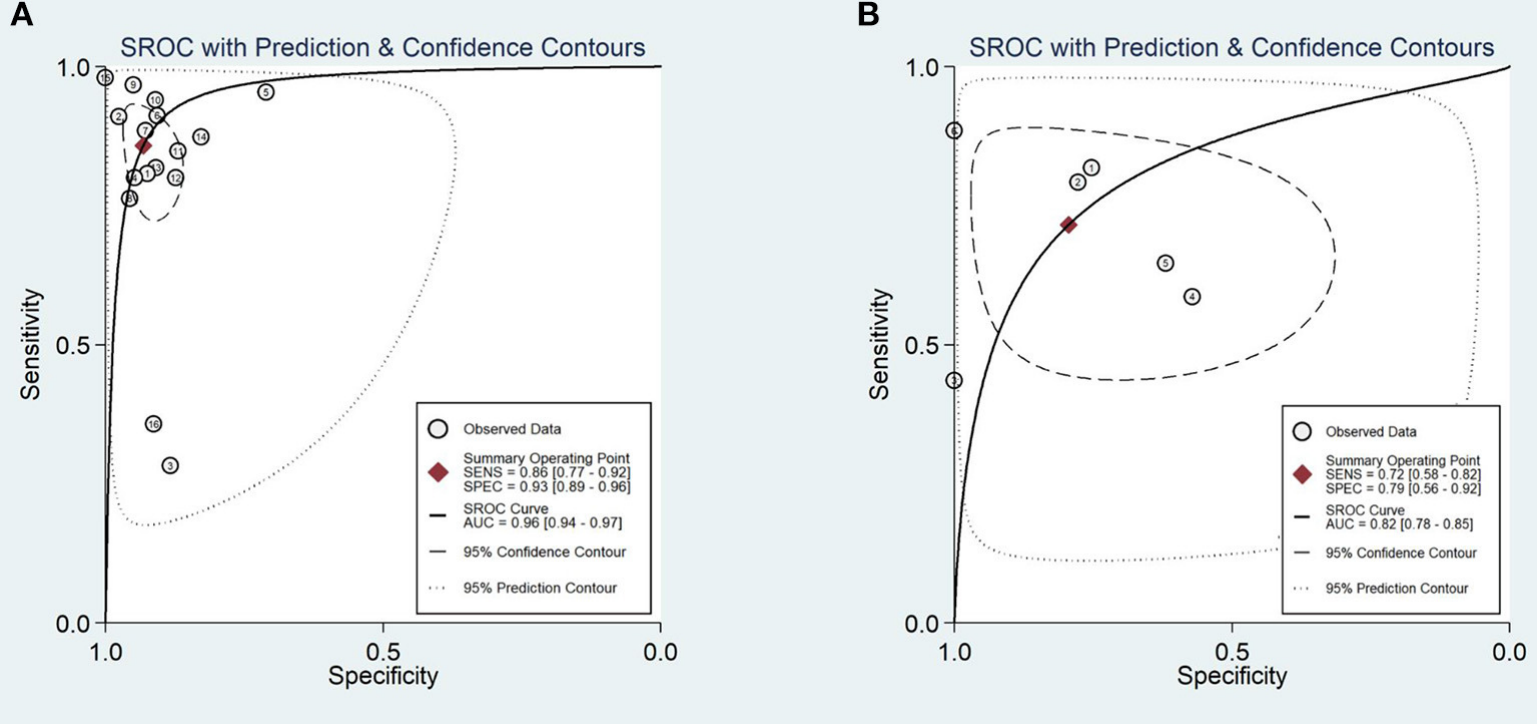
Area under the receiver operating characteristic curve **(A)**. The AUC of the AI-assisted endoscopy diagnose in the EGC detection was 0.96 (95% CI, 0.94–0.97). **(B)** The AUC of the AI-assisted endoscopy diagnose in the EGC depth distinction was 0.82 (95% CI, 0.78–0.85).

A total of 170,8519 images were utilized for machine training. A total of 22,621 EGC images from the 16 studies were included in the meta-analysis of EGC diagnosis. The diagnostic ability of AI-assisted endoscopy in each study is shown in Supplementary Table 1. The AUC of the AI-assisted endoscopy diagnosis in EGC detection was 0.96 (95% CI, 0.94–0.97) with heterogeneity I^2^ value of 0.98, thus the random effect model was applied. The pooled sensitivity was 86% (95% CI, 77–92%), and the specificity was 93% (95% CI, 89–96%). While the AUC, sensitivity and specificity of AI-assisted depth distinction was 0.82 (95% CI, 0.78–0.85), 72% (95% CI, 58–82%), and 79% (95% CI, 56–92%). The forest plots of sensitivity, specificity of AI detection and depth distinction are shown in Figures 2, 3. ROC of detection and depth distinction are shown in Figure 4. Influence analysis showed that Bum-Joo Cho, Hiroya Ueyama, and Yusuke Horiuchi's study had the greatest impact on the results (Supplementary Figure 3). After rejecting them, the pooled AUC, sensitivity and specificity were 0.95 (95% CI, 0.93–0.97), 85% (95% CI, 78–90%), and 92% (95% CI, 90–94%), respectively, which still indicated an accurate diagnostic ability of AI-aided diagnosis of EGC. The funnel plot asymmetry with a *p*-value of 0.81 showed the absence of publication bias for the included studies (Supplementary Figure 4).

### Other Factors That Have an Impact on the Accuracy of AI

The effects of the original images from WLE or NBI on the AI diagnostic ability were compared. The sensitivity of the NBI image application was 95% (95% CI, 91–97%), while that of WLE was 73% (95% CI, 57–85%), and the specificity was 96% (95% CI, 70–100%) and 93% (95% CI, 90–95%).

When the number of training images was more than 10,000, the sensitivity and specificity were 88% (95% CI, 83–92%) and 94% (95% CI, 91–96%), respectively, more than that of the sensitivity 85% (95% CI, 69–93%) and specificity 93% (95% CI, 82–97%) of the group that had >10,000 training images.

**Figure 5:**
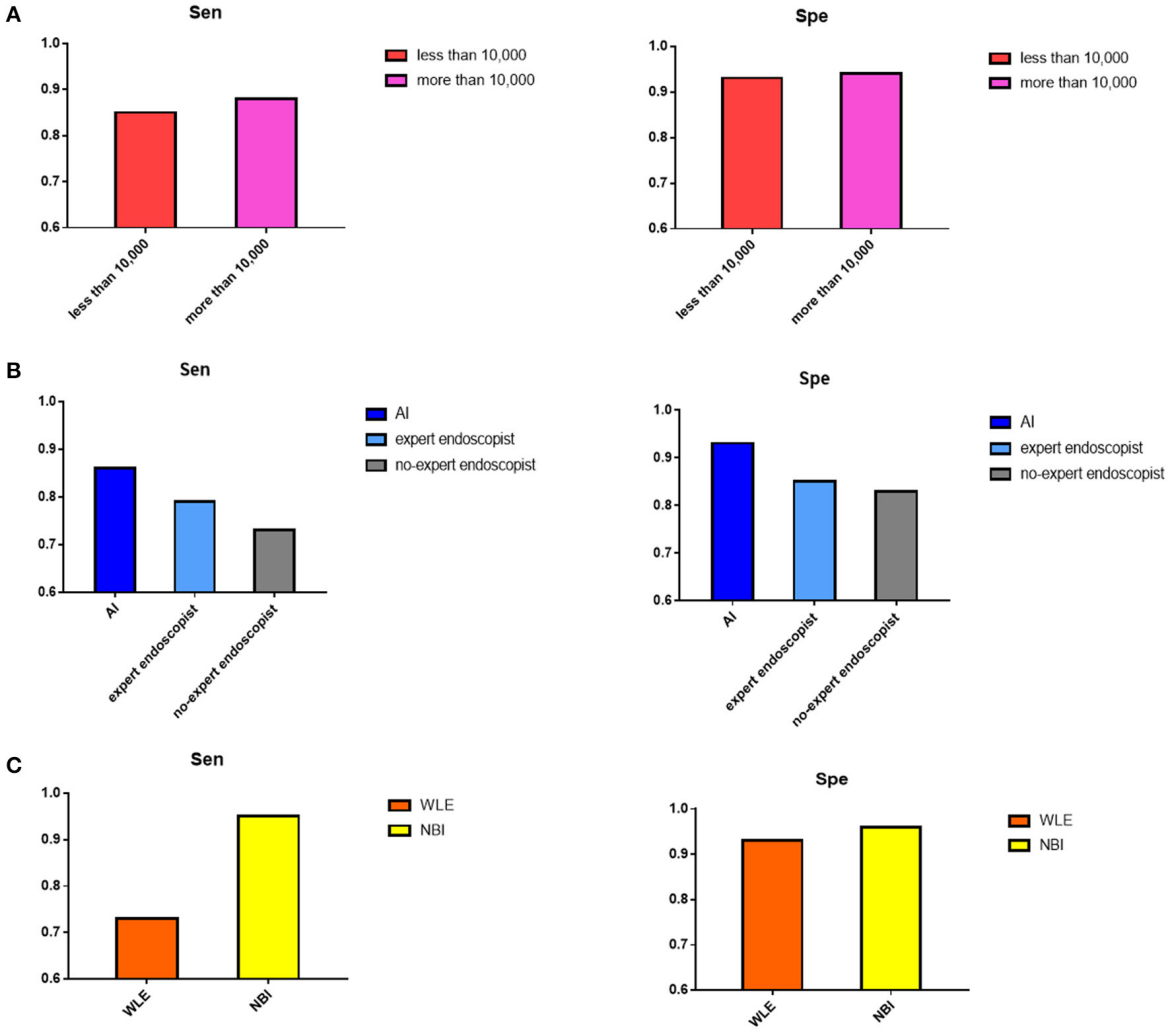
Result of subgroup analysis. **(A)** The pooled sensitivity and specificity of number of images in training process showed when the images were more than 10,000, the diagnostic value would be better. **(B)** The pooled sensitivity and specificity of AI detection, expert endoscopist, and non-expert endoscopist showed AI detection and expert endoscopist judgement were significantly more accurate than non-expert endoscopist. **(C)** The pooled sensitivity and specificity of original images extracted by NBI and WLE showed NBI image applied performed better.

For the control group, sensitivity and specificity of the expert endoscopist vs. non-expert endoscopist diagnosis were 79% (95% CI, 61–90%) vs. 73% (95% CI, 61–82%), 85% (95% CI, 77–90%) vs. 83% (95% CI, 67–92%), respectively. Here, the general expert endoscopists were those who had clinical experience with endoscopy examination for more than 10 years. Figure 5 shows the subgroup results.

## Discussion

Japanese researchers published a minimum required standard for the “systematic screening protocol for the stomach,” which comprised 22 images of the stomach to precisely discover suspicious cancerous lesions (42). In 2016, the European Society of Gastrointestinal Endoscopy (ESGE) published a protocol comprising 10 images of the stomach (43). However, these protocols could not be carried out absolutely, and endoscopists may miss some regions during the examination due to individual operative levels and subjective factors, which can lead to the misdiagnosis of EGC (44–46).

Deep learning (47, 48), which is typically based on artificial neural networks, aims at learning multilevel manifestations of data to make predictions. The development of deep convolutional neural networks has particularly altered the computer vision field (49, 50).

Application of AI recognition with endoscopic images to detect the depth of wall invasion of gastric cancer was initially reported by Keisuke Kubota with an accuracy of 64.7% (51). Soon afterwards, several studies have shown excellent results for advanced technology. Hence, it is necessary to summarize the existing studies to realize the probable ability of AI on EGC detection and discuss what factors may influence the results.

This is the first meta-analysis on the performance of AI on EGC diagnosis with endoscopy. In this article, we indicated that the application of AI in endoscopic detection of EGC achieved an AUC of 0.96 (95% CI, 0.94–0.97), a sensitivity of 86% (95% CI, 77–92%), and a specificity of 93% (95% CI, 89–96%), which manifested a more accurate diagnostic ability than independent detection by endoscopists, while the depth distinction was dissatisfied with a sensitivity, specificity and AUC of 0.82 (95% CI, 78–85%), 72% (95% CI, 58–82%), and 79% (95% CI, 56–92%). The common reasons for misdiagnosis were lesions of gastritis or flat or depressed texture and anatomical structure which was hard to identify. The cancer invasion depth was classically distinguished by morphologically evaluating several findings such as the concentration of stomach wall folds, the marginal ridge, the elasticity and thickness of the lesion, and the presence of variant of the stomach wall due to the volume of insufflation air in the stomach with WLE (52–54). Furthermore, the accuracy of discriminating EGC depth by conventional endoscopy was reported to be 62–80% (55). Thus, the AI applied endoscopy performed well on EGC depth determination. Bum-Joo Cho, Hiroya Ueyama and Yusuke Horiuchi's study (23, 26, 40) showed significant heterogeneity. Cho et al. used the Inception-Resnet-v2 model with an AUC of 74.5 (95% CI, 67.9–80.4) and a sensitivity of 28.3 (95% CI, 16.0–43.5). The included poor-quality images, composition of the database, and pathological classification criteria may cause poor diagnostic performance. In addition, we performed several subgroup analyses to delineate the probable influencing factors of AI performance.

For the algorithm model, Simonyan et al. (56) investigated the value of the convolutional network depth on its accuracy in large-scale image recognition setting. The result showed that when the depth was pushed to 16–19 weight layers, it would have a significant improvement on the prior-art configurations. VGG-16 had 16 convolutional and three fully connected layers, which were carried out by five max-pooling layers and used filters with a small receptive field to achieve a low error rate in practice. On the other hand, SVM also performed excellently in the included studies. SVM is utilized in distinguishing two classes and creating the boundary line to maximize the distance between the hyperplane and the nearest sample. Compared to other mathematical models (57–59), SVMs are utilized to model physical systems by adapting their parameters (60–63). SVMs are widely known for their application in classification (64).

The endoscopic image modality of validation set should be same to the training set. For training images from different endoscopy modalities, the sensitivity of studies using images from NBI seemed to be better than those using images from WLE (96 vs. 93%). A model which was trained with NBI images could only recognize NBI images in practice. However, a multicenter randomized controlled trial that compared a non-magnifying NBI with WLI indicated no significant difference in gastric cancer detection (65). Although NBI is currently regarded as the most broadly applied image-enhanced modality in AI research, the impact of other imaging modalities, such as the lately available linked-color imaging or blue-laser imaging modalities, need more studies for verification.

For the number of training images, it seemed that the more images the machine trained, the more accurate the AI detection would be. The concept that a large number of images are a prerequisite to structure a learning model was also certified in the research conducted by Seguí et al. (66) for motility movement classification in wireless capsule endoscopy. A recent meta-analysis similarly indicated that a ten-fold increase in training data size could improve the accuracy of AI detection by 3% (67).

Neural networks have the potential capacity for clinical practice and can be significantly popularized in the gastrointestinal field. However, CNN detection is temporarily in the stage of research. This study also had some limitations. A limited number of available studies fit the inclusion criteria since the novel technology has just been developed in recent years. Thus, the subgroup results were not completely reliable due to the limited number of studies. All the included studies were retrospective, which may lead to selection bias of included images, particularly in the validation dataset. In addition, few studies provided a solution to multiple gastrointestinal abnormalities as comparison, while most studies only researched the detection of a single abnormality, including Barrett's esophagus, Helicobacter pylori infection, early gastric cancer, atrophic gastritis, etc. (68–70), which is insufficient for clinical application. Moreover, an AI EGC detection model based on full-length videos was scarce, which postpones its general application in clinical practice.

To overcome these limitations, several projects can be carried out in the future. More prospective studies can be designed for strict images, including criteria, high-definition image extraction and expert endoscopist involvement to prove higher level evidence. Luo et al. (71) has carried out a multicenter, case-control, prospective real-time diagnostic study on artificial intelligence for detection of esophagus and gastric cancer with accuracy of 0.955 (95% CI 0.952–0.957). GRAIDS algorithm, which was based on the concept of DeepLab's V3+ (72, 73), was utilized in this prospective study. Expanding the training image number is necessary to improve the machine recognition ability. On the other hand, the validation images are supposed to be larger. Training images extracted from different endoscopy modalities still need to be investigated to establish a popularized dataset. Currently, limited data have shown that the VGG-16, SSD, and SVM classifier models are credible computer-aided diagnosis algorithms. Another branch of deep learning, deep reinforcement learning (DRL), recently performed at the top level in the GO game in 2016 (74). DRL is likely to be applied in the EGC detection field. DRL combines deep learning with reinforcement learning, incorporating not only the excellent perception and distinguishing abilities of deep learning in visual tasks but also the decision-making capabilities of reinforcement learning (75). DRL has performed well in dealing with dynamic decision problems (74–76). However, DRL has not yet been used in clinical trials. Wu et al. (77) reported that the application of WISENSE, a mechanism that utilizes aspects of both CNN and DRL, could decrease the number of blind spots during an upper endoscopy, initially achieving an accuracy of 90.02%. The exploration of accurate algorithms is worthy of being explored.

## Conclusion

This is the first meta-analysis to summarize current evidence of AI applications in EGC diagnosis. The AI applications seemed to be more accurate in parts of EGC detection than the endoscopists. The VGG-16, SSD, and SVM classifier models probably performed better according to the limited studies. When the number of training images is expanded, the accuracy will be improved. More strictly designed perspective studies with different reliable CNN algorithms are needed to make AI universal in clinical practice.

## Data Availability Statement

The datasets presented in this study can be found in online repositories. The names of the repository/repositories and accession number(s) can be found in the article/Supplementary Material.

## Author Contributions

KJ: study concept and design and analysis and interpretation of data. XJ and YW: acquisition of data. SW, KN, ZZ, SJ, and PLiu: literature search. XJ, JP, and YH: figure processing. PLi and SL: critical revision of the manuscript for important intellectual content. FL: study supervision. All authors contributed to the article and approved the submitted version.

## Conflict of Interest

The authors declare that the research was conducted in the absence of any commercial or financial relationships that could be construed as a potential conflict of interest.

## Supplementary Material

Supplementary Figure 1 Literature screening flow according to PRISMA.

Supplementary Figure 2 Quality of included studies according to QUADAS-2 scale.

Supplementary Figure 3 Influence analysis showed the significantly heterogeneity in Bum-Joo Cho, Hiroya Ueyama, and Yusuke Horiuchi's study.

Supplementary Figure 4 Funnel plot showed no publication bias among the included studies.

## Abbreviations

EGC: Early gastric cancer
AUC: Area under the receiver operating characteristic curve
ROC: Receiver operating characteristic
WLI: White light imaging
WLE: White light endoscopy
NBI: Narrow band imaging
BLI: blue-laser imaging
EMR: Endoscopic mucosal resection
ESD: Endoscopic submucosal dissection
WHO: World Health Organization
AI: Artificial intelligence
CNN: Convolutional Neural Network
CAD: Computer aided detection
CIs: Confidence intervals
VGG-16: Visual Geometry Group-16
SSD: Single Shot MultiBox Detector
SVM: Support vector machines
DRL: Deep reinforcement learning
Grad-CAM: gradient-weighted class activation mapping.
