# Corrigendum: Current Evidence and Future Perspective of Accuracy of Artificial Intelligence Application for Early Gastric Cancer Diagnosis With Endoscopy: A Systematic and Meta-Analysis

**DOI:** 10.3389/fmed.2021.698483

**Published:** 2021-05-14

**Authors:** Kailin Jiang, Xiaotao Jiang, Jinglin Pan, Yi Wen, Yuanchen Huang, Senhui Weng, Shaoyang Lan, Kechao Nie, Zhihua Zheng, Shuling Ji, Peng Liu, Peiwu Li, Fengbin Liu

**Affiliations:** ^1^First College of Clinic Medicine, Guangzhou University of Chinese Medicine, Guangzhou, China; ^2^Department of Spleen-Stomach and Liver Diseases, Traditional Chinese Medicine Hospital of Hainan Affiliated to Guangzhou University of Chinese Medicine, Haikou, China; ^3^Department of Gastroenterology, First Affiliation Hospital, Guangzhou University of Chinese Medicine, Guangzhou, China

**Keywords:** artificial intelligence, machine learning, deep learning, early gastric cancer, endoscopy

**The authors' names and surnames were accidentally inverted. The correct author list appears below:**

Kailin Jiang^1^, Xiaotao Jiang^1^, Jinglin Pan^2^, Yi Wen^3^, Yuanchen Huang^1^, Senhui Weng^1^, Shaoyang Lan^3^, Kechao Nie^1^, Zhihua Zheng^1^, Shuling Ji^1^, Peng Liu^1^, Peiwu Li^3^^*^ and Fengbin Liu^3^^*^

The authors apologize for this error and state that this does not change the scientific conclusions of the article in any way. The original article has been updated

