# Supplementary material for: Current Evidence and Future Perspective of Accuracy of Artificial Intelligence Application for Early Gastric Cancer Diagnosis With Endoscopy: A Systematic and Meta-Analysis
Source: Front Med (Lausanne). 2021 Mar 15;8:629080. doi: 10.3389/fmed.2021.629080 (PMC8005567; doi:10.3389/fmed.2021.629080)
Supplement: Supplementary file 1 [file Table_1.DOCX]

| Author | Year | Nation | Training image | Testing image | Algorithm | Sen（%） | Spe（%） | Expert Sen­­（%） | Expert Spe（%） | Non-expert Sen（%） | Non-expert Spe（%） | Observation | Comparison | Time |
| --- | --- | --- | --- | --- | --- | --- | --- | --- | --- | --- | --- | --- | --- | --- |
| Hong Jin Yoon | 2019 | Korea | 11539 | 2450 | VGG-16 | 80.7 | 92.5 | / | / | / | / | EGC | Chronic gastritis, chronic atrophic gastritis, intestinal metaplasia, and erosion | Not mentioned |
|  |  |  |  | 800 |  | 81.7 | 75.4 | / | / | / | / | T1a (mucosa) | T1b (submucosa) | Not mentioned |
|  |  |  |  | 2450 | VGG-16 based on Grad-CAM | 91.1 | 97.6 | / | / | / | / | EGC | Chronic gastritis, chronic atrophic gastritis, intestinal metaplasia, and erosion | Not mentioned |
|  |  |  |  | 800 |  | 79.2 | 77.8 | / | / | / | / | T1a (mucosa) | T1b (submucosa) | Not mentioned |
| Bum-Joo Cho | 2019 | Korea | 5017 | 200 | Inception-ResNet-v2 | 28.3 | 88.3 | / | / | 51.4 | 90.5 | EGC | Advanced gastric cancer/Dysplasia/Non neoplasm | 0.0264 seconds per image |
| Y. Sakai | 2018 | Japan | 348943 | 9650 | GoogLeNet | 80.0 | 94.8 | / | / | / | / | EGC | Non-cancer | Not mentioned |
| Yusuke Horiuchi | 2019 | Japan | 2570 | 258 | GoogLeNet | 95.4 | 71.0 | 80.5 | 79.3 | 75.9 | 75.6 | EGC | Gastritis | 0.02 seconds per image |
| Lan Li | 2019 | China | 20000 | 341 | Inception-v3 | 91.2 | 90.6 | 79.8 | 94.1 | 76.0 | 67.6 | EGC Lesions of mucosal high-grade neoplasia and submucosal invasion by carcinoma | non-cancerous lesions: negative for neoplasia, indefinite for neoplasia and mucosal low-grade neoplasia | Not mentioned |
| Toshiaki Hirasawa | 2018 | Japan | 13,584 | 2271 | SSD | 88.5 | 92.7 | / | / | / | / | T1a (mucosa); T1b (submucosa) | Gastritis; Hyperplastic polyp or other benign lesions | 0.02 seconds per image |
|  |  |  |  | 77 |  | 88.5 | 100 | / | / | / | / | T1a (mucosa); T1b (submucosa) | T2 (muscularis propria);  T3 (subserosa) ; T4a (serosa) | 0.02 seconds per image |
| Yan Zhu | 2019 | China | 632 | 203 | ResNet50 | 76.5 | 95.6 | 91.1 | 70.6 | 85.2 | 57.4 | EGC | SM2, Muscularis propria, Subserosa, Serosa | 0.18 seconds per image |
| Takashi Kanesaka | 2017 | Japan | 126 | 81 | SVM classifier | 96.7 | 95.0 | / | / | / | / | Depressed-type EGC ≤10 mm | Non-cancer | 0.41 seconds per image |
| Lianlian Wu | 2018 | China | 1.28 million | 200 | VGG-16, ResNet-50 | 94.0 | 91.0 | 94.0 | 87.0 | 84.0 | 82.5 | EGC | Normal, Superficial gastritis, Mild erosive gastritis | 230 milliseconds per frame |
| Rie Miyaki | 2013 | Japan | 493 | 92 | SVM | 84.8 | 87.0 | / | / | / | / | EGC | Non-cancer | Not mentioned |
| Yohei Ikenoyama | 2020 | Japan | 13,584 | 2806 | SSD | 0.8 | 0.873 | / | / | 53.3 | 97.2 | gastric cancer | non-neoplastic lesions/actual number of non-neoplastic lesions | 0.0154 s per image |
|  |  |  |  | 126 |  | 43.6 | 100 | / | / | / | / | T1a (mucosa) | T1b (submucosa) | 0.0154 s per image |
| Hussam Ali | 2018 | Pakistan | 176 | 176 | SVM on G2LCM descriptors | 91.0 | 82.0 | / | / | / | / | metaplasia and dysplasia | Non-cancer | Not mentioned |
| Bum-Joo Cho | 2020 | Korea | 2590 | 179 | Inception-ResNet-v2 | 58.7 | 57.2 | / | / | / | / | EGC invaded to mucosal | EGC invaded to Submucosal | Not mentioned |
|  |  |  |  | 179 | DenseNet-161 | 64.47 | 62.4 | / | / | / | / | EGC invaded to mucosal | EGC invaded to Submucosal | Not mentioned |
| Yusuke Horiuchi | 2020 | Japan | 2570 | 174 | GoogLeNet | 87.4 | 82.8 | / | / | / | / | Videos including cancerous parts during ESD | Videos including noncancerous parts during ESD | 0.033 s per frame |
| Hiroya Ueyama | 2020 | Japan | 5574 | 2300 | ResNet-50 | 98.0 | 100.00 | / | / | / | / | differentiated type EGC | non-cancerous mucosa or non-cancerous  lesions | 0.026 s per image |
| Tingsheng Ling | 2020 | Japan | 2217 | 1870 | VGG-16 and ResNet-50 | 88.6 | 78.6 | 71.8 | 80.0 | / | / | differentiated type EGC | undifferentiated type EGC | 25 frames per second |
| Liming Zhang | 2020 | China | 1121 | 237 | ResNet34 | 36.8 | 91.2 | 32 | 86.7 | / | / | EGC and high-grade intraepithelial  neoplasia | Lesion Free, Peptic ulcer, Advanced gastric cancer, submucosal tumor | 42 seconds to diagnose 1,091 endoscopic  images |

Supplement Table 1. Diagnostic ability of AI applied in EGC diagnosis. EGC: Early gastric cancer
