# Supplementary material for: Current Evidence and Future Perspective of Accuracy of Artificial Intelligence Application for Early Gastric Cancer Diagnosis With Endoscopy: A Systematic and Meta-Analysis
Source: Front Med (Lausanne). 2021 Mar 15;8:629080. doi: 10.3389/fmed.2021.629080 (PMC8005567; doi:10.3389/fmed.2021.629080)
Supplement: Supplementary Figure 1 — Literature screening flow according to PRISMA. [file Data_Sheet_1.pdf]

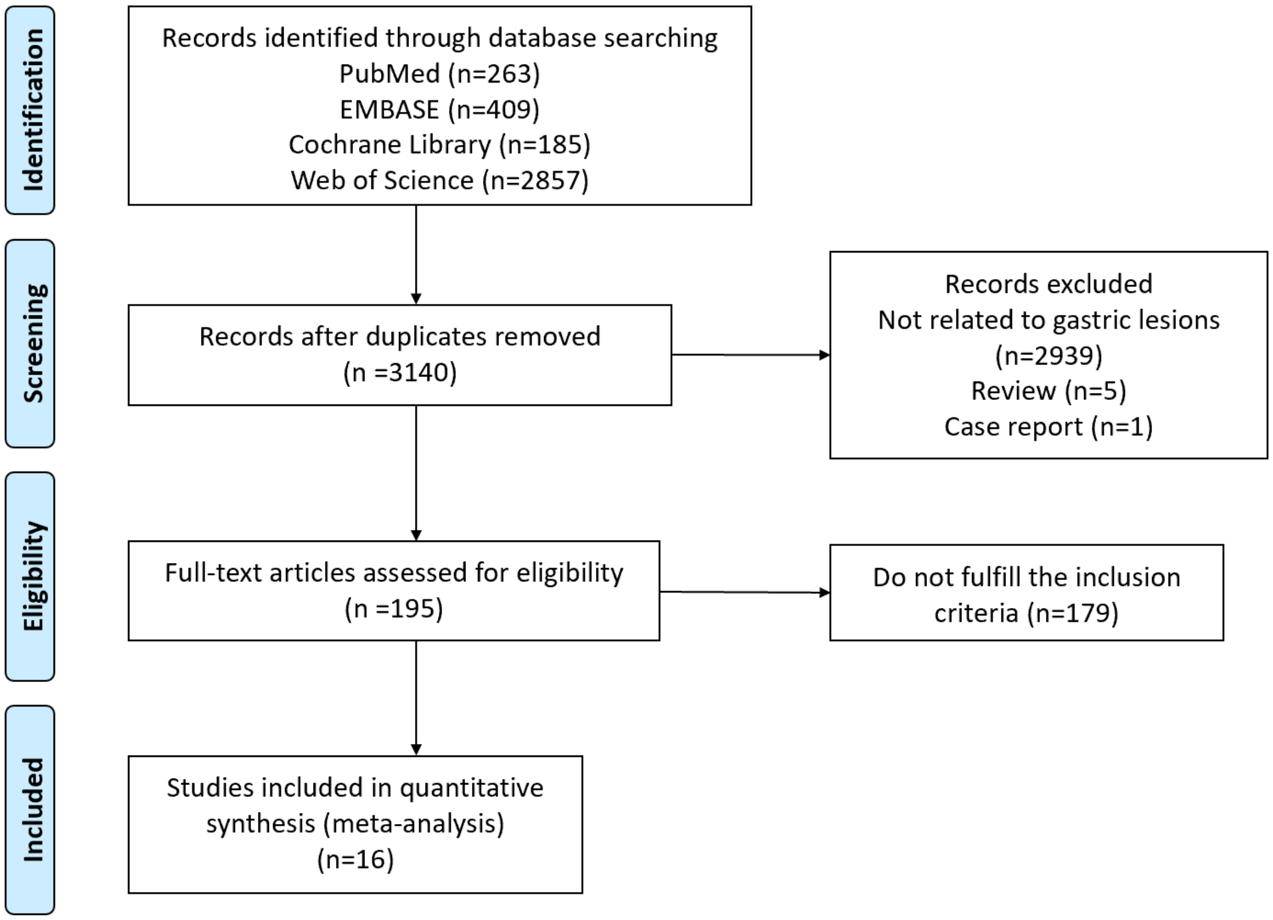

Supplemental Figure1. Literature screening flow according to PRISMA

Study	Year	Risk of Bias				Applicability Concerns		
		Patients Selection	Index Test	Reference Standard	Flow and Timing	Patients Selection	Index Test	Reference Standard
Hong Jin Yoon	2019	+	+	+	+	+	+	+
Bum-Joo Cho	2019	-	+	?	+	+	+	+
Y. Sakai	2018	+	+	?	+	+	+	?
Yusuke Horiuchi	2019	+	+	?	+	+	+	+
Lan Li	2019	+	+	+	+	+	+	+
Toshiaki Hirasawa	2018	-	+	+	+	?	+	+
Yan Zhu	2019	+	+	+	+	+	+	+
Takashi Kanesaka	2017	+	+	+	+	?	+	+
Lianlian Wu	2018	+	+	+	+	+	+	?
Rie Miyaki	2013	?	+	+	+	?	+	?
Yohei Ikenoyama	2020	+	+	+	+	+	+	?
Hussam Ali	2018	?	+	+	+	-	+	?
Bum-Joo Cho[18]	2019	+	+	+	+	+	+	+
Yusuke Horiuchi	2020	+	+	+	+	+	+	+
Hiroya Ueyama	2020	+	+	+	+	+	+	+
Liming Zhang	2020	?	+	+	+	-	+	+

Supplemental Figure 2. Quality of included studies according to QUADAS-2 scale.

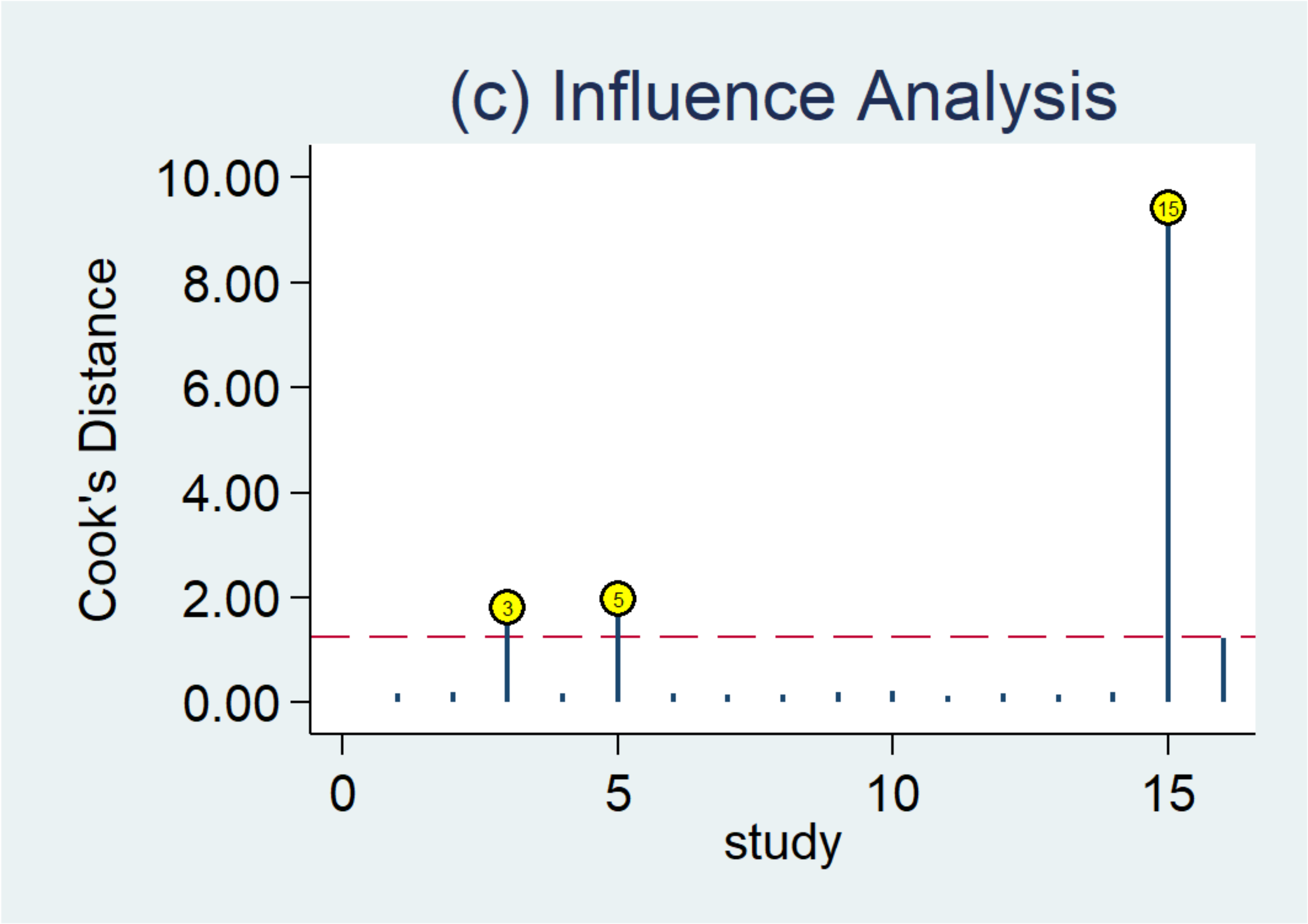

Supplemental Figure 3. Influence analysis showed the significantly heterogeneity in Bum-Joo Cho, Hiroya Ueyama and Yusuke Horiuchi’s study.

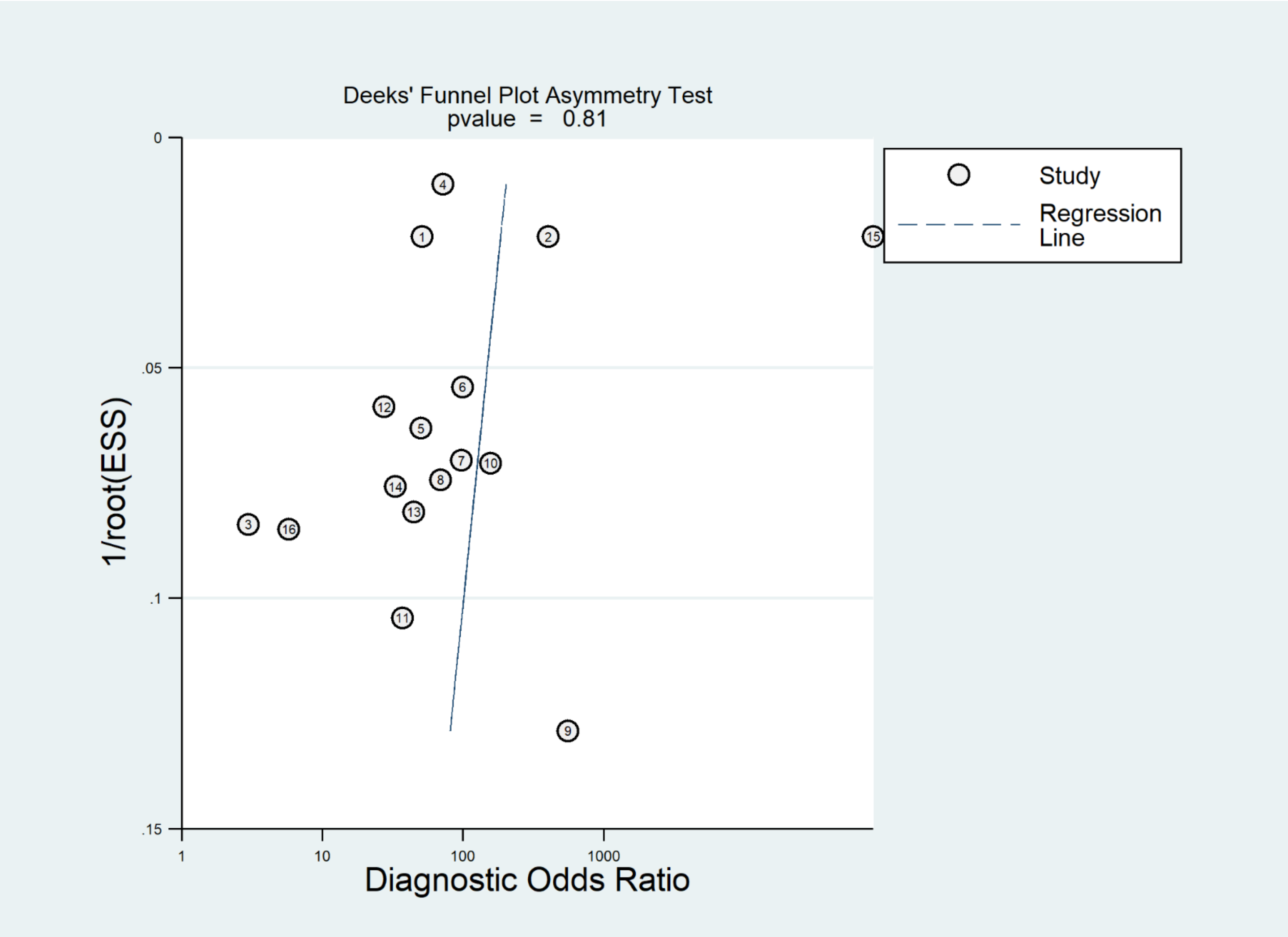

Supplemental Figure 4. Funnel plot showed no publication bias among the included studies.
